# Exosomes in osteoporosis: regulatory mechanisms and clinical applications

**DOI:** 10.3389/fphys.2026.1792752

**Published:** 2026-04-10

**Authors:** Qi Shuai, Jun Tian, Wenlong Yang, Santing Huang

**Affiliations:** 1 Department of Spine Surgery, Tongren Hospital of Traditional Chinese Medicine, Tongren, Guizhou, China; 2 Graduate School, Guizhou University of Traditional Chinese Medicine, Guiyang, Guizhou, China

**Keywords:** bone metabolism regulation, exosomes, intercellular communication, molecular mechanisms, osteoporosis, targeted therapy

## Abstract

Osteoporosis is a systemic metabolic bone disorder characterized by progressive bone mass reduction and microarchitectural deterioration, leading to fragility fractures. It poses a serious threat to the health of middle-aged and elderly populations and imposes a heavy healthcare burden. Exosomes mediate intercellular communication by transporting bioactive molecules such as proteins, miRNAs, and circRNAs, exerting bidirectional regulation on bone homeostasis. Exosomes derived from mesenchymal stem cells, young plasma, and plants often improve osteoporosis by promoting osteoblastic differentiation and suppressing osteoclastic activity. Conversely, exosomes originating from osteoclasts, M1 macrophages, and tumor cells tend to accelerate bone resorption. Exosomes not only provide highly specific non-invasive biomarkers for osteoporosis but also emerge as novel therapeutic carriers due to their inherent biocompatibility, targeted delivery properties, and potential for engineered modification. This systematic review examines the biological properties of exosomes, the molecular mechanisms by which exosomes from different sources regulate bone metabolism, and their application progress in the diagnosis and treatment of osteoporosis. It also explores challenges in their clinical translation, providing a comprehensive reference for further research and clinical application in this field.

## 1 Introduction

Osteoporosis is the most prevalent metabolic bone disease worldwide. According to the World Health Organization (WHO) criteria, it is diagnosed when the bone mineral density (BMD) T-score measured by dual-energy X-ray absorptiometry (DXA) is ≤ -2.5, or when there is a history of fragility fractures regardless of BMD value (Kanis, 1994). Globally, approximately one-third of women and one-fifth of men over the age of 50 will experience an osteoporotic fracture (Johnell and Kanis, 2006). Fractures not only cause severe pain and functional impairment but also incur associated medical, nursing, and socioeconomic costs reaching hundreds of billions of dollars annually. With the accelerating trend of global population aging, this burden is growing at an unprecedented rate (Burge et al., 2007). The fundamental pathophysiology of osteoporosis lies in an imbalance of bone remodeling processes. Specifically, osteoblast-mediated bone formation weakens, while osteoclast-mediated bone resorption becomes relatively enhanced or hyperactive. This ultimately leads to a net reduction in bone mass, deterioration of bone microarchitecture, and decreased bone strength (Manolagas, 2000).

Current drug strategies for treating osteoporosis have achieved some success, yet they remain subject to significant limitations that cannot be overlooked. The efficacy of basic calcium and vitamin D supplements in preventing fractures has long been controversial. A large-scale randomized controlled trial involving over 36,000 postmenopausal women demonstrated that supplementation with calcium and vitamin D did not significantly reduce fracture incidence (Jackson et al., 2006). Antiresorptive agents, such as bisphosphonates and denosumab, effectively increase bone mineral density and reduce fracture risk. However, they primarily function by inhibiting osteoclast activity, failing to fundamentally reverse already compromised bone microarchitecture. Long-term use may be associated with rare but serious side effects, including osteonecrosis of the jaw and atypical femoral fractures (Shane et al., 2014). On the other hand, anabolic agents like teriparatide, a recombinant human parathyroid hormone, powerfully stimulate new bone formation. However, their high cost, requirement for daily subcutaneous injections, and typical 24-month treatment cycle limitation significantly restrict their patient population and long-term efficacy (Neer et al., 2001). More critically, most existing systemic drug delivery strategies lack specific targeting capability for bone tissue. The widespread distribution of drugs throughout the body may trigger off-target effects and adverse reactions. Consequently, the medical community urgently needs to explore and develop safer, more effective, and bone-targeted novel therapeutic strategies to address the increasingly severe challenge of osteoporosis.

Exosomes, as a research hotspot in intercellular communication, present new opportunities for the diagnosis and treatment of osteoporosis. Exosomes are a type of membrane-bound vesicle actively secreted by nearly all types of eukaryotic cells into the extracellular environment, typically ranging in diameter from 30 to 150 nanometers (Théry et al., 2002). Their biogenesis begins with the formation of early endosomes through the coordinated action of the endoplasmic reticulum and Golgi apparatus within the cell. These early endosomes further mature into multivesicular bodies (MVBs) containing multiple intraluminal vesicles (ILVs). Ultimately, MVBs fuse with the plasma membrane, releasing the ILVs as exosomes (Xia et al., 2025). These minute vesicles are widely distributed in nearly all bodily fluids, including blood, lymph fluid, urine, and cerebrospinal fluid, forming a complex intercellular information network (Valadi et al., 2007). The core function of exosomes lies in their cargo, which includes specific proteins such as membrane transporters, heat shock proteins, and cytoskeletal proteins; Abundant nucleic acids including microRNAs, mRNAs, long non-coding RNAs, circular RNAs, and DNA fragments; And unique lipid components (Mathivanan et al., 2010). These bioactive molecules are protected by the exosome’s lipid bilayer membrane, enabling stable circulation in bodily fluids and targeted internalization by recipient cells. This facilitates precise signal transmission from donor cells to recipient cells, regulating physiological or pathological processes (Raposo and Stoorvogel, 2013).

In the field of bone metabolism research, the role of exosomes is gaining increasing attention. Acting as messengers within the bone tissue microenvironment, they efficiently shuttle between osteoblasts, osteoclasts, osteocytes, mesenchymal stem cells (MSCs), and immune cells. By delivering bioactive molecules they carry, exosomes profoundly influence the proliferation, differentiation, and function of these cells (Urabe et al., 2018). Research indicates that the regulatory effects of exosomes exhibit high source dependency and target specificity. Exosomes secreted by MSCs from young, healthy individuals are typically rich in osteogenic factors, whereas those from aged or pathologically compromised cells, such as osteoclasts or tumor cells, may carry signals that inhibit bone formation or promote bone resorption (Weilner et al., 2016). Compared to direct stem cell-based therapies, exosomes offer multiple advantages as an acellular therapeutic extremely low immunogenicity, high biocompatibility, ease of crossing biological barriers, physicochemical stability, and potential for engineered modifications to enhance targeting and therapeutic efficacy (Lai et al., 2015). These characteristics not only position exosomes as promising carriers for precision drug delivery in osteoporosis but also render their unique contents in bodily fluids novel biomarkers for early disease diagnosis and prognosis monitoring.

This paper aims to systematically review and summarize the latest advances in exosome research in the field of osteoporosis. We provide a detailed overview of the fundamental biological characteristics of exosomes, including their biogenesis, internalization pathways, cargo-sorting mechanisms, and current mainstream isolation and purification techniques. We then conduct an in-depth analysis of how exosomes from different cellular sources regulate bone metabolic homeostasis via specific molecular mechanisms, with a particular focus on their roles in the pathological processes of osteoporosis. We also highlight the application potential and cutting-edge developments of exosomes in the diagnosis, treatment, and fracture repair of osteoporosis. In addition to summarizing recent progress, this review critically discusses the existing controversies, technical bottlenecks, and challenges in clinical translation, with the goal of providing an objective and in-depth reference for future studies. Finally, we outline the key challenges and future directions in this field, aiming to establish a solid theoretical basis and comprehensive reference for promoting the clinical translation of exosome-based research from bench to bedside.

## 2 The biology of exosomes

### 2.1 The biogenesis of exosomes

**Figure 1:**
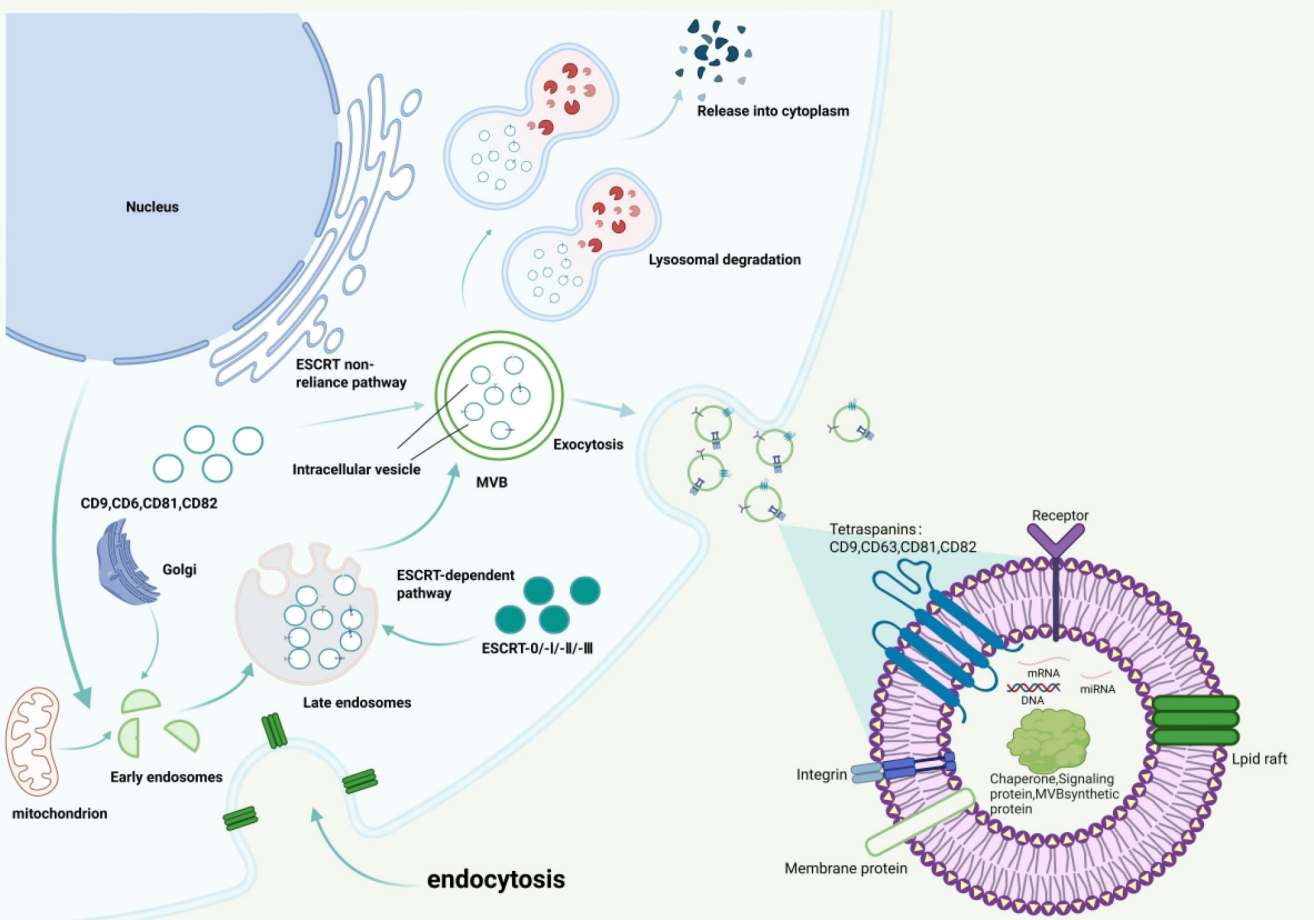
Schematic diagram of exosome biogenesis and release mechanisms. ① Initiation stage: The cell membrane engulfs extracellular components or intracellular biomolecules via endocytosis, forming early sorting endosomes (ESE). ESEs can fuse with the endoplasmic reticulum or the trans-Golgi network (TGN) to enrich their contents;② Maturation stage: ESEs further develop into late sorting endosomes (LSEs). Mediated by the ESCRT complex, LSE membranes invaginate to encapsulate bioactive molecules (mRNA, miRNA, proteins, lipids), forming intraluminal vesicles (ILVs). LSEs containing ILVs then become multi-vesicular bodies (MVBs). ③ Fate Determination: MVB can fuse with lysosomes for degradation and recycling of contents, or undergo cytoskeletal transport to the plasma membrane regulated by RAB GTPases; ④ Release Stage: MVB dock and fuse with the plasma membrane via SNARE complexes, releasing ILVs through exocytosis to form exosomes with diameters of 40–150 nm. The lipid bilayer structure of exosomes protects their biomolecular cargo from enzymatic degradation, establishing the structural foundation for their subsequent applications as diagnostic biomarkers and therapeutic carriers. ESCRT = Endosomal Sorting Complex, Transport and Recycling.

Exosome formation is a tightly regulated multi-step process, initiated by plasma membrane invagination to generate early endosomes, which can fuse with the endoplasmic reticulum (ER) or trans-Golgi network (TGN) for cargo enrichment (Colombo et al., 2014). Early endosomes mature into late endosomes, whose membranes invaginate to form ILVs; Late endosomes containing ILVs are defined as MVBs. MVBs have two fates, fusion with lysosomes for cargo degradation, or migration to the plasma membrane to release ILVs as 40–150 nm exosomes via exocytosis (Kowal et al., 2014). This process relies on two core pathways: the ESCRT-dependent pathway, a classical mechanism wherein four ESCRT subcomplexes and auxiliary proteins mediate the recognition of ubiquitinated cargo, as well as membrane invagination and cleavage, to form ILVs (Babst, 2011), and the ESCRT-independent pathway driven by tetraspanins (CD9, CD63, CD81, CD82) and lipid molecules (Andreu and Yáñez-Mó, 2014). The biogenesis and release mechanism is illustrated in (**Figure 1**).

In addition to the classical ESCRT-dependent pathway and ESCRT-independent pathway mediated by tetraspanins and ceramides, recent studies have identified multiple non-traditional ‘budding’ pathways involved in exosome biogenesis. These include: RAB31-FLOTs-specific pathway independent of ESCRT and responsible for sorting specific membrane protein cargo (Chang et al., 2024); nuclear envelope budding pathway where exosome precursors directly bud from the nuclear membrane and carry nuclear contents such as DNA fragments (Arya et al., 2022); direct plasma membrane budding pathway where exosomes are directly released from the plasma membrane under stress conditions (Nabhan et al., 2012); atypical ESCRT pathway relying on partial ESCRT components to simplify the budding process (Babst, 2011). These non-traditional pathways synergize with classical pathways to form a diverse exosome biogenesis network, and the exosomes generated thereby may carry unique bioactive cargo that could indirectly affect bone metabolic balance by regulating intercellular communication in the bone microenvironment. However, the specific roles of these non-traditional pathway-derived exosomes in osteoporosis remain to be further explored. The syndecan-syntenin-ALIX complex also fine-tunes cargo sorting and exosome secretion efficiency (Shakhova et al., 2012). Whose lipid bilayer structure protects exosomal cargo from enzymatic degradation, laying the foundation for its diagnostic and therapeutic applications. Notably, the functional differences between exosomes derived from traditional and non-traditional pathways remain unclear. For example, whether exosomes from the nuclear envelope budding pathway carry unique bone metabolism-related nucleic acids or proteins has not been verified.

### 2.2 Exosome internalization

After extracellular release, exosomes are transported systemically via blood/lymph or mediate local paracrine/autocrine signaling through direct cell-cell contact (Tkach and Théry, 2016). They interact with recipient cells in two core modes: (1) Surface ligands, growth factors and cytokines, bind to recipient cell receptors to activate downstream signaling without exosomal entry; (2) Content delivery via membrane fusion or endocytosis, the primary regulatory mode which alters the recipient cell’s gene expression and functional state (Valadi et al., 2007). Exosome internalization is cell type-dependent, with six major pathways: macropinocytosis, clathrin-mediated endocytosis, caveolae-mediated endocytosis, lipid raft-mediated endocytosis, phagocytosis in specialized phagocytes, and direct membrane fusion (Mulcahy et al., 2014). Although some internalized exosomes are degraded in lysosomes, recipient cells exhibit significant functional changes, confirming an efficient endosome escape mechanism that ensures cargo-mediated signal transmission (Feng et al., 2010).

### 2.3 Exosome cargo

Exosomes are hailed as microcosms of cells, yet their contents are not randomly packaged cytoplasmic components. Instead, they undergo a highly selective and actively regulated sorting process that ensures the specificity of their functions. The sorting mechanism for proteins is the most complex. The sorting of transmembrane proteins primarily relies on the ESCRT system recognizing the ubiquitinated modifications of their cytoplasmic domains (Stuffers et al., 2009). Soluble proteins may be incorporated into ILVs through association with these transmembrane proteins. Additionally, other post-translational modifications such as SUMOylation and phosphorylation have been demonstrated to regulate the selective enrichment of specific proteins like ALIX and heterogeneous nuclear ribonucleoprotein A2B1 (hnRNPA2B1) (Villarroya-Beltri et al., 2013). Heat shock proteins (HSPs) may mediate the folding and loading of specific proteins through their molecular chaperone functions. Exosomes are rich in various small RNAs, particularly miRNAs. miRNA sorting involves specific sequence motifs. The EXO-motif, such as GGAG, can be recognized by hnRNPA2B1, mediating the loading of miRNAs containing this motif into exosomes (Kapinas and Delany, 2011). Y-box binding protein 1 (YBX1) is another key RNA-binding protein that can also recognize and regulate the exosomal sorting of various RNA molecules. Recent studies have also revealed that RNA chemical modifications, such as m6A methylation, participate in regulating RNA stability and its enrichment within exosomes (T. Chen et al., 2015). DNA, including double-stranded DNA (dsDNA), single-stranded DNA (ssDNA), and mitochondrial DNA (mtDNA), has also been detected in exosomes. Their sources and sorting mechanisms remain under investigation. In tumor cells, DNA fragments released from ruptured abnormal cytoplasmic micronuclei may be packaged into exosomes through interactions with the tetraspanin membrane protein family. This process is considered a mechanism for intercellular transmission of tumor gene mutation information (Thakur et al., 2014).

These carefully packaged contents collectively form the functional core of exosomes. In bone metabolism regulation, miRNAs (miR-214-3p, miR-26a) carried by exosomes can modulate osteoblast or osteoclast differentiation and function by targeting and suppressing the mRNA of key bone metabolism transcription factors (Runx2, Osterix) or signaling pathway components (PTEN, SMAD1) (D. Li et al., 2016). Annexins can directly bind calcium and phosphate, serving as a mineralization core to promote the mineralization process of the bone matrix (Rilla et al., 2014). Meanwhile, circular RNAs (circRNAs) participate in regulating the dynamic balance between bone formation and resorption by acting as miRNA sponges, competitively binding specific miRNAs to indirectly upregulate target gene expression (T. Li et al., 2013). These molecules collectively mediate the complex and precise regulation of bone homeostasis by exosomes.

Despite advances in sorting mechanisms, there are still unresolved controversies. For instance, the EXO-motif GGAG mediated by hnRNPA2B1 is considered a key signal for miRNA loading, but some osteogenic miRNAs such as miR-26a-5p lack this motif yet are highly enriched in MSCs-derived exosomes, suggesting the existence of uncharacterized sorting signals. Additionally, the crosstalk between different cargo sorting pathways such as protein ubiquitination and RNA m6A methylation remains unclear, and whether they coordinate to regulate exosome function in bone metabolism requires further investigation.

### 2.4 Exosome isolation methods

The isolation and purification of high-quality exosomes form the foundation for all downstream research and clinical applications. Due to the complex composition of bodily fluids, exosomes must be distinguished from cells, cellular debris, apoptotic bodies, protein aggregates, and lipoproteins. Currently, multiple separation techniques have been developed, each with distinct advantages and limitations ultracentrifugation (UC) serves as the gold standard method for exosome isolation. Its fundamental workflow involves a series of density gradient centrifugation steps: low-speed centrifugation (300×g) removes cells, medium-speed centrifugation (2,000×g) removes apoptotic bodies, high-speed centrifugation (10,000-20,000×g) removes large vesicles, and finally, UC (100,000-120,000×g) precipitates exosomes. To enhance purity, sucrose or iopamidol density gradient centrifugation is often incorporated. This method is well-established and reliable but is time-consuming and labor-intensive, requiring expensive UC equipment. It may cause mechanical damage to exosomes and yields relatively low quantities, making it difficult to adapt for high-throughput clinical sample processing (Théry et al., 2006). Ultrafiltration (UF) utilizes membranes with a molecular weight cutoff typically of 100 kDa or pore sizes of 0.1-0.22 μm. Through centrifugation or pressure, small-molecule proteins and solvents pass through while larger particles like exosomes are retained on the membrane. This method is simple and rapid, effectively preserving exosome bioactivity and structural integrity. However, membrane clogging is common, and non-specific adsorption may cause sample loss (Fang et al., 2024). Size exclusion chromatography (SEC) separation based on particle size. The sample passes through a column packed with porous gel microspheres. Large particles like exosomes cannot enter the microsphere pores and rapidly elute via the shortest path, while small particles enter the pores, taking a longer path and eluting more slowly. SEC effectively removes soluble protein contamination, preserves the natural structure and functional activity of exosomes, and offers good reproducibility (Böing et al., 2014). Its main drawback is the difficulty in completely separating exosomes from lipoproteins of similar size. In recent years, separation efficiency has significantly improved through optimized column packing materials and coupled techniques (Gámez-Valero et al., 2016). Polymer-based precipitation adds macromolecules like polyethylene glycol (PEG) to samples, displacing water molecules, reducing exosome solubility and inducing precipitation. This method is extremely simple to operate, requires no specialized equipment, and is suitable for processing large sample volumes. However, it yields substantial co-precipitated protein and lipoprotein contaminants, resulting in the lowest purity among all methods and is generally not recommended for functional studies. Immunoaffinity capture utilizes magnetic beads or columns coated with antibodies targeting exosome surface markers (CD9, CD63, CD81) to specifically capture exosomes. This method achieves extremely high purity and can isolate specific exosome subpopulations from designated cellular sources. However, it is costly, yields low output, and may introduce bias in captured exosome populations due to the selection of specific markers. Microfluidics integrates sample processing, separation, and detection functions onto micrometer-scale chips. Separation principles can be based on size, acoustic waves, electric field properties, or affinity capture. Microfluidics offers distinct advantages including minimal sample consumption, rapid processing, high automation, and scalability for high-throughput operations. It is particularly well-suited for rapid, standardized exosome isolation and analysis of small clinical samples (C. Chen et al., 2010). The comparison results of different exosome isolation methods have been summarized in **Table 1**.

**Table 1:** Summary and comparison of different exosome isolation methods.

Separation methods	Advantages	Disadvantages	References
UC	1. Technologically mature and reliable, serving as the “gold standard”; 2. Isolated exosomes exhibit high integrity; 3. Can be combined with density gradient centrifugation to enhance purity.	1. Time-consuming and labor-intensive, requiring expensive ultracentrifuges; 2. High-speed centrifugation may cause mechanical damage to exosomes; 3. Low yield, unsuitable for high-throughput clinical samples.	(Afrisham et al., 2025; Dhar et al., 2025; Mussin et al., 2025)
UF	1.Simple and rapid operation; 2. Preserves exosome biological activity and structural integrity effectively; 3. Suitable for rapid sample pretreatment.	1. Filter membranes are prone to clogging; 2. Non-specific adsorption occurs, leading to sample loss; 3. Separation purity is moderate.	(He et al., 2019; Tembo et al., 2025)
SEC	1. Effectively removes soluble protein contamination; 2. Maximizes preservation of exosome natural structure and functional activity; 3. Excellent reproducibility; 4. Separation efficiency can be enhanced by optimizing packing material.	1. It is difficult to completely separate exosomes from lipoproteins of similar size; 2. Further purification using other methods is required.	(Bai et al., 2023; Kapoor et al., 2024)
Polymer-based precipitation	1. Simple operation, no special equipment required; 2. Low cost; 3. Suitable for processing large-volume samples.	1. Lowest purity, prone to coprecipitation with impurities such as proteins and lipoproteins; 2. Suitable only for preliminary enrichment; not recommended for functional studies.	(Huang et al., 2025; Marzouk et al., 2025)
Immunoaffinity capture method	1. High purity; 2. Capable of specifically capturing exosome subpopulations from designated cell sources; 3. Suitable for targeted functional studies.	1. High cost; 2. Low yield; 3. Antibody specificity may introduce bias in the captured population.	(Din et al., 2024; JLi et al., 2025)
Microfluidic technology	1. Extremely low sample consumption; 2. Rapid processing speed; 3. High degree of automation enabling high-throughput capabilities; 4. Suitable for rapid standardized separation of small clinical samples.	1. High technical barriers; 2. High chip manufacturing costs; 3. Currently primarily in laboratory research and development, not yet fully mainstream.	(J. Li et al., 2025; Xing et al., 2025; Zidong Zhou et al., 2025)

With continuous technological advancements, strategies combining multiple methods alongside novel techniques such as tangential flow filtration and acoustic fluid separation, based on new materials and principles are emerging. These innovations aim to standardize, enhance efficiency, and automate the exosome isolation and purification process, thereby clearing technical hurdles for exosome clinical translation.

## 3 Functional mechanisms of exosomal cargo in osteoporosis

Exosomes exert bidirectional regulatory effects on bone homeostasis primarily through their selectively sorted bioactive cargo, including miRNAs, proteins, circRNAs, and long non-coding RNAs (lncRNAs). These molecules are the direct mediators of intercellular communication between osteoblasts, osteoclasts, MSCs, and immune cells, thereby participating in the pathogenesis and progression of osteoporosis. Below is a systematic elaboration of the core cargo components, their diverse cellular sources, and regulatory mechanisms in bone metabolism, integrating all original research evidence while emphasizing the central role of cargo in mediating exosomal function.

### 3.1 miRNAs as core regulators of osteogenic/osteoclastic differentiation

MiRNAs are the most extensively studied exosomal cargo in osteoporosis, with distinct expression profiles across cellular sources and clear targeting mechanisms. They primarily regulate bone metabolism by suppressing the expression of key transcription factors or signaling pathway components, and their functional diversity is closely associated with the cellular origin of exosomes.

Osteoclast-derived exosomes (OC-EVs) during RANKL-induced osteoclast differentiation, OC-EVs are significantly enriched with miR-214-3p. Upon internalization by osteoblast precursor cells or mature osteoblasts, this miRNA directly targets and suppresses the expression of ATF4, a key osteogenic transcription factor, thereby inhibiting osteoblast activity and matrix mineralization, and reducing bone formation (D. Li et al., 2016). Clinical studies confirm that serum miR-214-3p levels are significantly elevated in elderly women at high fracture risk, and inhibiting miR-214-3p effectively mitigates bone loss in ovariectomized (OVX) mouse models. In contrast, OC-EVs also contain miR-324, which targets ARHGAP1 a negative regulator of osteogenic differentiation, promoting the osteogenic differentiation of MSCs and reflecting the dual functional potential of OC-EV-derived miRNAs (M. Liang et al., 2021).

M1 macrophage-derived exosomes (M1-EVs) carry miR-98 and miR-155. Among them, miR-98 continuously activates the JNK pathway by targeting DUSP1, inhibiting MSC osteogenic differentiation and exacerbating postmenopausal osteoporosis (Yu et al., 2021). Myoblast-derived exosomes are associated with the functional coupling of muscle and bone, and sarcopenia frequently coexists with osteoporosis. Exosomes secreted by myoblasts contain miR-196a-5p, which directly inhibits osteoclast formation, forming a functional complement between muscle and bone (Takafuji et al., 2021). Mesenchymal stem cell-derived exosomes (MSC-EVs) are a rich source of osteogenic miRNAs. Human bone marrow MSC-EVs deliver miR-186, which targets Mob1, a key inhibitor of the Hippo signaling pathway, activating YAP/TAZ and promoting the expression of downstream osteogenic genes (Runx2, Osterix) to enhance bone formation in OVX rats. Adipose-derived MSC-EVs carry miR-335-3p, which improves osteoporosis by targeting the Aplnr axis (Sheng et al., 2022). Hypoxia-pretreated MSC-EVs exhibit elevated miR-126 levels, which activate the Ras/ERK pathway in endothelial cells by targeting SPRED1, promoting angiogenesis and creating a favorable microenvironment for bone repair (W. Liu et al., 2020).

Urine-derived stem cell exosomes (USCs-EVs) have shown remarkable potential in ameliorating diabetic osteoporosis (DOP). As a severe complication of diabetes, DOP is characterized by bone microstructural damage and decreased bone quality induced by a hyperglycemic environment, thereby increasing the risk of fractures (X. Zhang et al., 2025). USCs-EVs are rich in miR-26a-5p, a microRNA that exerts a key regulatory role by being delivered to osteoprogenitor cells. Its molecular mechanism lies in the fact that miR-26a-5p directly targets and inhibits the expression of histone deacetylase 4 (D. Zhang et al., 2023). Subsequent silencing of HDAC4 relieves its inhibitory effect on downstream signaling pathways and activates the hypoxia-inducible factor-1α (HIF-1α)/vascular endothelial growth factor A (VEGFA) signaling axis, ultimately achieving effective intervention in diabetic osteoporosis DOP. Serum-derived exosomes are rich in miR-3960, whose expression level is significantly downregulated in the serum of osteoporosis patients. As a positive regulator of osteogenic differentiation, miR-3960 typically promotes Runx2-mediated osteogenic differentiation by targeting and inhibiting Hoxa2, a negative regulator of osteogenesis. In pathological states, the reduced activity of this pathway leads to a decrease in circulating miR-3960, which serves as a promising non-invasive biomarker for the early screening of osteoporosis (Shao et al., 2020).

Osteoblast-derived exosomes (OB-EVs) under physiological conditions, OB-EVs carry miR-503-3p, which targets and downregulates heparanase (Hpse) expression in osteoclast precursor cells, inhibiting osteoclast differentiation and function to form a negative feedback regulatory loop for bone resorption (Q. Wang et al., 2021a). However, mature osteoblasts also secrete small vesicle subpopulations (-400 nm) enriched with miR-143-3p. This miRNA targets Cbfb mRNA, simultaneously inhibiting osteoblastic differentiation and stimulating osteoclast formation, thereby exacerbating bone loss (Uenaka et al., 2022).

Exosomes secreted by breast cancer cells, MDA-MB-231 cells in particular, are abundant in miR-20a-5p and miR-17-5p, which synergistically disrupt the balance of bone metabolism through distinct mechanisms. Specifically, exosomal miR-20a-5p can be internalized by bone marrow-derived macrophage precursors and directly targets and inhibits the expression of SRCIN1 Src kinase signaling inhibitor 1, thereby relieving its suppressive effect on osteoclast differentiation. This directly promotes the proliferation and differentiation of osteoclasts and enhances bone resorptive activity. On the other hand, miR-17-5p may inhibit osteoblast activity by targeting osteogenesis-related signaling molecules, for example the BMP pathway, leading to a reduction in bone formation. Ultimately, this imbalance between bone resorption and bone formation induces osteolytic bone destruction (Guo et al., 2019). The functional descriptions of exosomes from different sources have been summarized in **Table 2**.

**Table 2:** Functional descriptions of exosomes from different sources.

Donor cells/source	Stimulus/ condition	Cargo	Receptor cell	Function description	References
Osteoclasts	RANKL-induced differentiation	miR-214-3p	Osteoblast precursor cells/Mature osteoblasts	Targeted inhibition of ATF4 expression, a key osteogenic transcription factor, reduces osteoblast activity and matrix mineralization capacity, thereby decreasing bone formation.	(D. Li et al., 2016)
Osteoclasts	–	lncRNA AW011738	Osteoblasts	Sponge adsorption of miR-24-2p releases its post-transcriptional suppression of TREM1, ultimately inhibiting osteoblast differentiation and accelerating osteoporosis progression.	(J. Liu et al., 2024)
Osteoclasts	–	miR-324	MSCs	ARHGAP1, a negative regulator of osteogenic differentiation, promotes osteogenic differentiation of MSCs.	(M. Liang et al., 2021)
Osteoblasts (physiological state)	–	miR-503-3p	Osteoclast precursor cells	Targeted downregulation of Hpse expression inhibits osteoclast differentiation and function, thereby maintaining bone homeostasis.	(Q. Wang et al., 2021a)
Osteoblasts (Osteoporosis)	–	Circ_0008542	Osteoclasts	m6A methylation enhances the sponge-binding capacity of miR-11-185p, upregulates expression of the target gene RANK, and promotes osteoclast generation and bone resorption.	(Wang et al., 2021b)
Mesenchymal stem cells (adipose-derived)	–	miR-335-3p	BMSCs	Targeting the Aplnr axis to improve osteoporosis	(Sheng et al., 2022)
Mesenchymal stem cells	–	Telomerase-associated active molecules	BMSCs	Restore telomerase activity in bone marrow-derived mesenchymal stem cells (BMSCs) to rejuvenate their microenvironment-regulating functions and improve bone density.	(Sonoda and Yamaza, 2023)
Mesenchymal stem cells (adipose-derived)	–	NLRP3 inflammasome inhibitor	Osteoclasts	Inhibits NLRP3 inflammasome activation in osteoclasts, reduces secretion of proinflammatory cytokines IL-1β and IL-18, and alleviates excessive bone resorption.	(L. Zhang et al., 2021a)
Mesenchymal stem cells	Hypoxia pretreatment	miR-126	endothelial cells	Targeting SPRED1 activates the Ras/ERK pathway, promoting endothelial cell proliferation, migration, and luminal formation, thereby enhancing the microvascular network within bone tissue.	(W. Liu et al., 2020)
M1-type macrophages	Inflammation/Infection Environment	miR-155、miR-98	MSCs	miR-98 targets DUSP1 to continuously activate the JNK pathway, inhibiting osteogenic differentiation of MSCs and exacerbating bone loss in postmenopausal osteoporosis.	(Yu et al., 2021)
Macrophage	Mechanical force stimulation	UCHL3	BMSCs	Promotes SMAD1 phosphorylation and nuclear translocation, activates the BMP/SMAD signaling pathway, and enhances the osteogenic differentiation of BMSCs.	(P. Pu et al., 2023)
endothelial cells	–	miR-155	Osteoblasts	Inhibiting osteoblast ferroptosis reverses massive osteoblast death in glucocorticoid-induced osteoporosis	(R. Z. Yang et al., 2021)
endothelial cells	–	lncRNA NEAT1	Macrophage	Promote macrophage polarization toward the M2 phenotype, improve the bone immune microenvironment, and indirectly promote bone formation.	(R. Z. Yang et al., 2021)
Myoblasts	–	miR-196a-5p	Osteoclasts	Directly inhibits osteoclast formation	(Takafuji et al., 2021)
Myoblasts	–	Prrx2	BMSCs	Regulating the lncRNA-MIR22HG/YAP pathway promotes osteogenic differentiation of BMSCs and alleviates osteoporosis.	(Y. Li et al., 2023)
Tumor cells (breast cancer)	Tumor Microenviro-nment	miR-20a-5p	Osteoclasts	Targeting SRCIN1 promotes osteoclast proliferation and differentiation, leading to osteolytic bone destruction.	(Guo et al., 2019)
Urine-derived stem cells	–	CTHRC1、OPG	Osteoblasts, osteoclasts	Enhances osteoblast activity while inhibiting osteoclast bone resorption, thereby bidirectionally regulating bone metabolism.	(C. Y. Chen et al., 2019)
Urine-derived stem cells	–	miR-26a-5p	Osteoblasts, osteoclasts	Through the HDAC4/HIF-1α/VEGFA axis, improve diabetic osteoporosis by enhancing osteogenesis and inhibiting osteoclast activity.	(D. Zhang et al., 2023)
Milk	–	miRNAs Bioactive peptides, miRNAs	Osteoblasts	Promotes osteoblast proliferation and differentiation, demonstrating potential for improving osteoporosis in mouse models.	(Go et al., 2021)
Akkermansia muciniphila	–	Exosome-like vesicles	MSCs, osteoclasts	Promoting MSC osteogenic differentiation and inhibiting osteoclast activation to regulate bone homeostasis through the gut-bone axis	(Gong et al., 2025)
Chinese Yam	–	Exosome-like nanovesicles	Osteoblasts, osteoclasts	Stimulates osteoblast proliferation, inhibits osteoclast differentiation, and improves osteoporosis.	(Hwang et al., 2023)

### 3.2 Proteins structural and functional mediators of bone metabolism

Exosomal proteins directly participate in bone formation, mineralization, and resorption, with functions closely related to their cellular sources. They act as enzymes, receptors, or signaling molecules to regulate bone cell activity, and their functional specificity is a key basis for exosome-mediated bone metabolism regulation. OB-EVs are rich in calcium-binding proteins such as annexins and alkaline phosphatase (ALP), key functional proteins for bone mineralization. Annexins directly bind calcium and phosphate ions, serving as nucleation centers to concentrate these ions in the extracellular matrix, initiating and promoting hydroxyapatite crystal formation. ALP, as a core enzyme in bone mineralization, further accelerates the mineralization process of the bone matrix, synergistically enhancing bone formation (Davies et al., 2017). Urinary stem cell-derived extracellular vesicles are rich in two key proteins, collagen triple helix repeat containing 1(CTHRC1) and osteoprotegerin (OPG), which achieve bidirectional regulation of bone metabolism through a synergistic effect. CTHRC1, a downstream molecule of the BMP2 signaling pathway, enhances osteoblast activity and maintains bone formation by promoting the proliferation and differentiation of osteoprogenitor cells and upregulating the expression of osteogenesis-specific genes. OPG, acting as a decoy receptor for RANKL, competitively blocks the binding of RANKL to RANK, thereby inhibiting the differentiation, survival and activation of osteoclasts and reducing bone resorption (C. Y. Chen et al., 2019). Milk-derived exosomes are rich in bioactive peptides that effectively promote the proliferation and differentiation of osteoblasts. They accelerate osteoblast proliferation by upregulating cell cycle-related proteins (CDK2, CDK4, Cyclin D1/E), and simultaneously induce the expression of the key osteogenic transcription factors RUNX2 and Osterix to facilitate osteogenic differentiation and mineralization (Go et al., 2021). Beyond their direct action on osteoblasts, milk-derived exosomes can also inhibit RANKL-induced osteoclast differentiation and indirectly improve bone metabolism by regulating the gut microbiota, which embodies the regulatory role of the gut-bone axis (Yun et al., 2020). MSC-EVs carry telomerase-associated active molecules, which restore telomerase activity in bone marrow-derived MSCs, once telomerase activity is restored, the recipient MSCs themselves will release exosomes carrying a variety of active molecules. These exosomes promote osteogenic differentiation through the downstream lncRNA MALAT1/SATB2 pathway, enhance osteogenic proliferation via the miR-935/STAT1 pathway, and dual inhibit RANKL-mediated osteoclastic activation through OPG and miR-21-5p (Sonoda and Yamaza, 2023). OC-EVs contain large vesicles (>100 nm) that are abundant in RANK receptors, a key protein cargo. These RANK-carrying exosomes bind to RANKL on the surface of osteoblasts, activating RANKL reverse signaling to promote osteoblast differentiation, a crucial complementary mechanism in bone remodeling coupling whereby osteoclasts summon osteoblasts for bone repair after completing bone resorption (Cao, 2018; Ikebuchi et al., 2018; Q. Ma et al., 2019). Tumor cell-derived exosomes carry proteins that cooperate with miRNAs to promote osteoclast proliferation and differentiation. For example, breast cancer cell-derived exosomes reshape the bone microenvironment and facilitate osteolytic destruction through the synergistic action of miR-20a-5p and protein mediators. miR-20a-5p is highly expressed in exosomes derived from MDA-MB-231 cells, and it inhibits the expression of SRCIN1 by directly targeting the 3’ untranslated region of SRCIN1 mRNA. As an inhibitor of Src kinase signaling, the downregulation of SRCIN1 relieves the inhibitory effect on osteoclastic differentiation, thereby promoting the proliferation and differentiation of bone marrow-derived macrophages. Meanwhile, various protein mediators carried by breast cancer exosomes form a synergistic regulatory network with miR-20a-5p. Protein mediators such as RANKL and M-CSF directly activate osteoclastic differentiation signaling pathways NF-κB, MAPK, creating a favorable microenvironment for the targeted regulation of miR-20a-5p (Guo et al., 2019). Exosomes derived from adipose-derived mesenchymal stem cells (AD-MSCs-Exos) improve diabetic osteoporosis by inhibiting the NLRP3 inflammasome. After intervention with AD-MSCs-Exos, miRNA bioactive molecules carried by exosomes target osteoclasts, suppress NLRP3 expression at the transcriptional level, or reduce the assembly and activation of the NLRP3 inflammasome by regulating the NF-κB pathway. The expression levels of NLRP3, caspase-1 p10, and IL-1β p17 in osteoclasts are significantly downregulated, accompanied by decreased secretion of IL-1β and IL-18. This inhibitory effect further attenuates the expression of osteoclast differentiation-related genes (NFATc1, c-Fos, TRAP) and reduces osteoclastic bone resorption activity, ultimately restoring bone mineral content and bone mineral density in a rat model of STZ-induced diabetic osteoporosis (L. Zhang et al., 2021a). Macrophage-derived exosomes are secreted by macrophages stimulated by mechanical force and contain ubiquitin carboxy-terminal hydrolase L3 (UCHL3), which promotes SMAD1 phosphorylation and nuclear translocation in bone marrow mesenchymal stem cells (BMSCs), activating the BMP/SMAD signaling pathway to enhance BMSC osteogenic differentiation (Panjun Pu et al., 2023). Exosomes derived from M2-type macrophages (M2-Exos) regulate bone metabolism by delivering IL-10 mRNA. M2-Exos are rich in IL-10 mRNA, and after being taken up by BMSCs and bone marrow-derived macrophages (BMDMs), they can be translated into functional IL-10 protein in recipient cells. As a key anti-inflammatory protein mediator, IL-10 initiates bidirectional regulatory signals by binding to the cell surface IL-10 receptor. In BMSCs, IL-10 activates the JAK1/STAT3 pathway, upregulates the expression of the key osteogenic transcription factors Runx2 and Osterix, and promotes osteogenic differentiation and mineralization. In BMDMs, IL-10 inhibits the NF-κB and MAPK pathways, downregulates the expression of the key osteoclast transcription factors NFATc1 and c-Fos, and suppresses osteoclast differentiation and bone resorption activity (Xutao. Chen et al., 2022).

### 3.3 CircRNAs/lncRNAs as miRNA sponges and epigenetic regulators

CircRNAs and lncRNAs in exosomes regulate bone metabolism primarily by acting as miRNA sponges, indirectly upregulating target gene expression and balancing bone formation and resorption. Their stability and specificity make them potential diagnostic biomarkers and therapeutic targets for osteoporosis. OB-EVs from osteoporosis patients exhibit significantly elevated Circ_0008542 levels. This circRNA undergoes m6A methylation modification, which enhances its capacity to sponge miR-11-185p. The sequestration of miR-11-185p upregulates the expression of its downstream target gene RANK, promoting osteoclast generation and bone resorption activity (W. Wang et al., 2021b). Serum exosomal hsa_circ_0006859 is significantly upregulated in osteoporosis patients, with an area under the receiver operating characteristic curve (AUC) value of up to 0.897 for the diagnosis of postmenopausal osteoporosis, indicating high sensitivity and specificity. As a competing endogenous RNA (ceRNA), hsa_circ_0006859 sponges miR-431-5p to relieve its inhibitory effect on the target gene ROCK1 mRNA, resulting in upregulated expression of ROCK1 protein. As a key signaling protein mediator, ROCK1 exerts bidirectional effects on the differentiation fate of BMSCs by regulating cytoskeleton remodeling and transcription factor activity. On the one hand, it inhibits the activity of the key osteogenic transcription factor RUNX2 and downregulates the expression of osteogenesis-related proteins (ALP, OCN, Col1a1); on the other hand, it promotes the activity of the key adipogenic transcription factor PPARγ and upregulates the expression of adipogenesis-related proteins (PPARγ, C/EBPα, FABP4) (Zhi et al., 2021). OC-EVs regulate osteoblast differentiation by delivering lncRNA AW011738. As a ceRNA, AW011738 acts as a molecular sponge for miR-24-2-5p, thereby relieving the translational inhibition of its target gene TREM1 mRNA by miR-24-2-5p, and ultimately leading to upregulated expression of TREM1 protein. The upregulation of TREM1 can suppress the expression of the key osteogenic transcription factors Runx2 and Osterix, and reduce ALP activity and mineralized nodule formation (J. Liu et al., 2024). Exosomes derived from endothelial cells regulate macrophage M2 polarization, improve the osteoimmune microenvironment, and indirectly promote osteogenesis by delivering lncRNA NEAT1. Exosomes secreted by human umbilical vein endothelial cells are rich in lncRNA NEAT1 and can be efficiently taken up by macrophages. After entering macrophages, NEAT1 inhibits the expression of DDX3X protein, thereby suppressing the assembly and activation of the NLRP3 inflammasome. Its downregulation reduces caspase-1 activation and decreases the maturation and secretion of IL-1β/IL-18, thus alleviating local inflammation. These signaling changes promote macrophage polarization toward the M2 phenotype with upregulated expression of CD206, IL-10, and Arg1 and inhibit M1 polarization with downregulated expression of CD86, IL-1β, and IL-6. M2-polarized macrophages indirectly promote the migration and osteogenic differentiation of BMSCs by secreting various soluble protein mediators (Y. Chen et al., 2023). Myoblast-derived exosomes regulate osteogenic differentiation of BMSCs and ameliorate osteoporosis by delivering the transcription factor Prrx2 protein. During myogenic differentiation, the expression of Prrx2 protein is upregulated in C2C12 myoblasts and packaged into exosomes for extracellular secretion. These exosomes can be efficiently internalized by BMSCs and deliver Prrx2 protein into the nucleus of recipient cells. As a transcription factor, Prrx2 directly promotes the transcription and expression of MIR22HG by binding to its promoter. As a ceRNA, MIR22HG sponges miR-128 to relieve the translational inhibition of its target gene YAP1 mRNA, resulting in upregulated expression and increased nuclear translocation of YAP1 protein. As a key effector of the Hippo signaling pathway, nuclear translocation of YAP1 activates the transcription of downstream osteogenic-related genes including RUNX2, Osterix, OCN, OPN and BMP2, thereby promoting osteogenic differentiation and mineralization of BMSCs (Y. Li et al., 2023).

### 3.4 Other functional cargo and synergistic regulation

Beyond the aforementioned categories, exosomes also carry bioactive molecules from natural sources including plants and probiotics, as well as lipids, which synergistically regulate bone metabolism, enriching the regulatory network of osteoporosis.

Exosome-like vesicles derived from Akkermansia muciniphila (Akk-EVs) regulate bone metabolism via the gut-bone axis (Yun et al., 2020). Akk-EVs can penetrate the intestinal barrier into the systemic circulation, target and accumulate in bone tissue, and be directly internalized by BMSCs and osteoclast precursors. In terms of osteogenic mechanisms, after entering BMSCs, protein components carried by Akk-EVs activate the BMP-2/Smad/Runx2 signaling axis: upregulating BMP-2 protein expression, inducing Smad1/5/8 phosphorylation, and promoting the expression of the key osteogenic transcription factors Runx2 and Osterix, ultimately enhancing ALP activity, mineralized nodule formation, and the synthesis of osteogenesis-related proteins (OCN, Col1a1); in terms of osteoclast-inhibiting mechanisms, upon uptake by osteoclast precursors, Akk-EVs suppress the NF-κB/MAPK/NFATc1 signaling axis: blocking RANKL-induced NF-κB phosphorylation, reducing the activities of the JNK/p38/ERK MAPK pathways, and inhibiting the expression of the key osteoclast transcription factors NFATc1 and c-Fos, thereby decreasing the formation of TRAP-positive multinucleated cells and bone resorption pit area. In addition, Akkermansia muciniphila can also synergize with Akk-EVs to regulate bone homeostasis through metabolic pathways including short-chain fatty acids SCFAs and vitamin K2, as well as immunomodulatory pathways including promoting Treg cell expansion and inducing M2 macrophage polarization (Gong et al., 2025).

**Figure 2:**
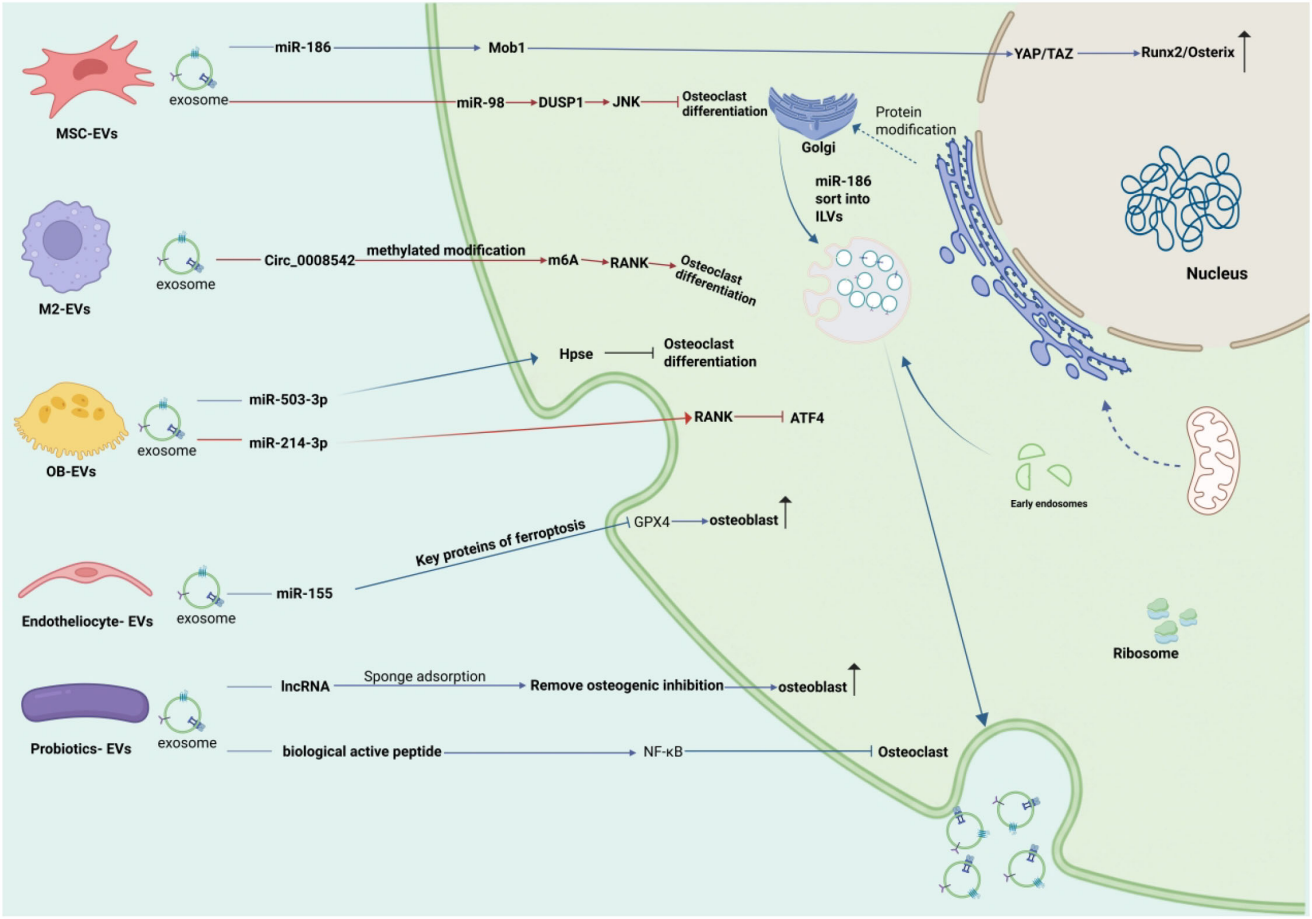
Regulatory mechanisms of exosomes from different sources in osteoporosis. OC-EVs, osteoclast-derived exosomes; OB-EVs, osteoblast-derived exosomes; MSC-EVs, mesenchymal stem cell-derived exosomes; M1-EVs, M1-type exosomes.

Yam-derived exosome-like nanovesicles (YNVs) promote osteogenic differentiation and ameliorate osteoporosis via the BMP-2/p-p38/Runx2 protein axis. After being taken up by MC3T3-E1 cells and primary osteoblasts, YNVs activate the expression of BMP-2 protein, which in turn induces the phosphorylation of p38 MAPK and upregulates the expression and transcriptional activity of the key osteogenic transcription factor Runx2. Runx2 promotes the synthesis of downstream osteogenic-related proteins, including ALP, OPN and COLI, thereby facilitating extracellular matrix mineralization (Hwang et al., 2023). The outline of the mechanism is depicted in (**Figure 2**).

## 4 Potential applications of exosomes in osteoporosis

### 4.1 Diagnostic tools

Given the pivotal role of exosomes in regulating bone metabolism and their unique biological characteristics, they demonstrate significant translational potential in the diagnosis and treatment of osteoporosis. Traditional osteoporosis diagnosis primarily relies on BMD measurements, yet BMD alone cannot fully reflect bone quality and fracture risk. As a liquid biopsy technology, exosomes dynamically reflect bone metabolic status through their contents, offering highly promising biomarkers for early, precise, and non-invasive osteoporosis diagnosis.

The miRNA profile in serum or plasma exosomes is the most extensively studied biomarker. As previously reported, miR-214-3p levels positively correlate with fracture risk in elderly women (D. Li et al., 2016). Multiple clinical studies have identified a series of differentially expressed miRNAs associated with osteoporosis through high-throughput sequencing. Levels of miR-766-3p and miR-1247-5p showed significant correlations with BMD values (Shi et al., 2022). A study in postmenopausal women found that serum exosomal miR-330-5p and miR-3124-3p levels were significantly higher in the osteoporosis group compared to healthy controls, with ROC curve analysis demonstrating good diagnostic performance. Another study constructed a diagnostic model incorporating miR-5330p and miR-5-3124p, effectively distinguishing postmenopausal osteoporosis patients from healthy individuals (Ciuffi et al., 2022). Proteomics analysis revealed specific protein biomarkers in serum exosomes from osteoporosis patients. Prefibrillin-1 exhibited elevated levels in serum exosomes from osteoporosis patients. A study employing quantitative proteomics and reverse engineering analysis identified a combination biomarker comprising four proteins,PSMB9, PCBP2, VSIR, and AARS, which achieved an AUC of 0.805 for diagnosing osteoporosis, demonstrating high diagnostic accuracy (Huo et al., 2019). CircRNAs, exceptionally stable due to their circular structure, serve as ideal biomarkers. Research revealed that hsa_circ_0006859 in serum exosomes was significantly upregulated in postmenopausal osteoporosis patients, exhibiting an outstanding diagnostic discrimination ability with an AUC value of 0.897 (Zhi et al., 2021). Furthermore, high-throughput sequencing of small RNAs analyzed the tRF expression profiles in plasma exosomes from 60 postmenopausal women, identifying 11 upregulated and 18 downregulated tRFs. Among these, tRF-25-R9ODMJ6B26, tRF-38-QB1MK8YUBS68BFD2, and tRF-18-BS68BFD2 as the three tRFs with the highest diagnostic value. ROC curve analysis of the constructed tRFs combination biomarker showed an AUC of 0.815, sensitivity of 76.7%, and specificity of 83.3%, suggesting potential as a supplementary marker for osteoporosis diagnosis (Y. Zhang et al., 2018).

Despite promising results from small-sample studies, exosomal miRNA biomarkers face significant challenges in clinical translation. First, the lack of unified detection standards. Different isolation methods such as UC and SEC lead to significant variation in miRNA expression levels. Second, the interference of comorbidities. Diabetes or chronic kidney disease, which are common in elderly osteoporosis patients, can alter serum exosomal miRNA profiles, leading to false positives. Third, single miRNA biomarkers have limited diagnostic efficiency, and multi-molecular combined models such as miRNA, circRNA and protein are needed to improve specificity, but such models are currently lacking large-sample multi-center validation.

Currently, blood samples are primarily used for detection due to their ease of acquisition and relatively abundant exosome content. Urinary exosomes, however, offer a completely non-invasive sampling method, making them particularly suitable for patients requiring long-term, dynamic disease monitoring, such as assessing treatment response (Huber et al., 2023). Urinary exosomes offer non-invasive sampling, but their cargo content is only one-fifth of that in serum exosomes, requiring high-sensitivity detection technologies such as digital PCR, which increases clinical costs. Additionally, urinary exosome composition is affected by hydration status and renal function, making it necessary to standardize sample collection conditions, a practice that has not been widely implemented in current research. Future trends will focus on developing diagnostic models based on multi-molecular combined detection, integrated with machine learning algorithms. This approach aims to further enhance diagnostic sensitivity and specificity, enabling early screening, classification diagnosis, and personalized risk assessment for osteoporosis (Huber et al., 2023).

### 4.2 Therapeutic drugs and engineering optimization

**Figure 3:**
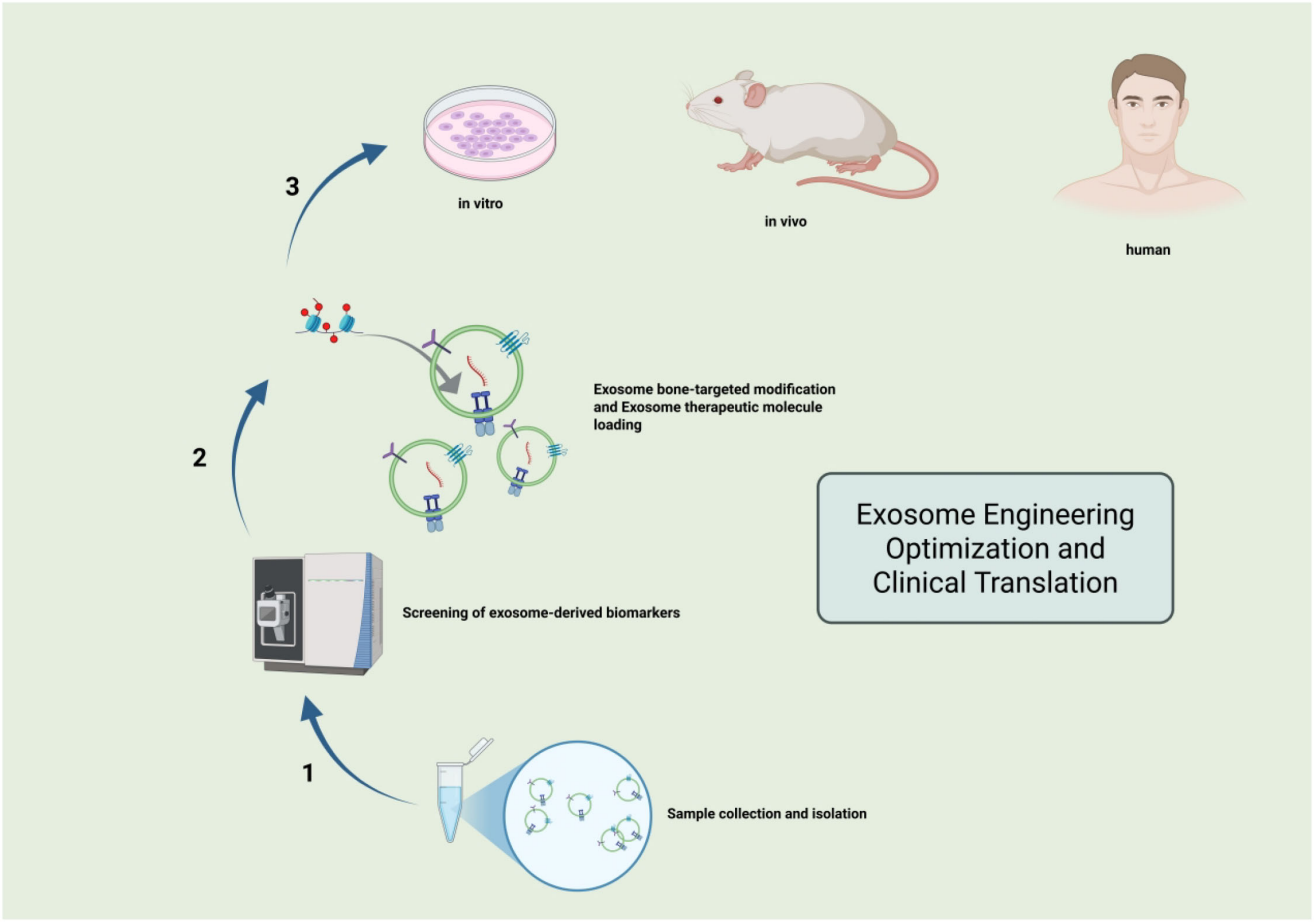
Schematic diagram illustrating the evaluation of engineered exosome optimization for osteoporosis treatment.

Exosomes, particularly MSC-EVs, have demonstrated significant potential as natural nanomedicines for treating osteoporosis in numerous animal studies. However, to enhance their clinical applicability, engineering modifications to overcome the limitations of natural exosomes are currently a key research focus. The advantages of natural exosomes are evident. Derived from autologous or allogeneic cells, they exhibit excellent biocompatibility and minimal immunogenicity, reducing the risk of immune rejection (Y. Yang et al., 2022); their lipid bilayer membrane structure protects internal bioactive substances from degradation by bodily enzymes, enabling prolonged half-lives in the bloodstream; and their nanoscale dimensions allow efficient penetration of vascular walls and even crossing of biological barriers like the blood-brain barrier to reach target tissues. Exosomes from specific sources exhibit natural chemotaxis toward injury or inflammation sites, accumulating in bone tissue to exert localized therapeutic effects (J. Wang et al., 2023). However, natural exosomes have inherent limitations despite their chemotaxis, most intravenously administered exosomes are rapidly cleared by reticuloendothelial system organs like the liver, spleen, and lungs, with only a limited proportion reaching bone tissue, thereby affecting therapeutic efficacy (F. Li et al., 2022); Therapeutic molecules in natural exosomes are typically present at low concentrations, potentially limiting their efficacy for moderate-to-severe osteoporosis. Furthermore, exosome yields are low, and variations in size, purity, and contents between batches hinder standardized, large-scale production (Shanshan et al., 2024). To address the issue of low exosome delivery efficiency, researchers have developed various surface modification strategies to equip them with navigation systems, guiding them to precisely reach bone tissue. The most common strategy involves covalently linking bone-targeting molecules, leveraging the exceptionally strong affinity of bisphosphonates for hydroxyapatite in the bone matrix. Through click chemistry or NHS-ester reactions, sodium alendronate molecules can be coupled to the amino or carboxyl groups of exosome membrane proteins, yielding bone-targeted exosomes (Gröger et al., 2014). *In vitro* experiments confirm that this modification significantly enhances the binding capacity of exosomes to hydroxyapatite. The outline of the mechanism is depicted in (**Figure 3**). Physical modification represents a milder non-covalent modification approach. For instance, bone-targeting peptides such as Asp-Ser-Ser and SDSSD peptides can specifically bind to DSPE-PEG-Peptide, a phospholipid derivative of periostin on the surface of osteoblasts. Co-incubation with exosomes utilizes the lipid insertion method to anchor the targeting ligand onto the exosome membrane. This method is operationally straightforward and causes minimal disruption to the natural structure and function of exosomes (Fan et al., 2020). One study successfully utilized SDSSD-modified exosomes to efficiently deliver Shn3-inhibiting siRNA to osteoblasts, thereby promoting bone formation (Cui et al., 2022). Genetic engineering involves modifying exosome-producing cells through genetic engineering to express specific targeting proteins on the membranes of secreted exosomes. For example, MSCs can be engineered to express the chemokine receptor CXCR4 via plasmid transfection. Since the ligand for CXCR4, stromal cell-derived factor-1 (SDF-1), is highly expressed in bone marrow, these secreted CXCR4-positive exosomes actively home to the bone marrow after intravenous injection, achieving highly efficient bone-targeting enrichment (Y. Hu et al., 2021). To enhance the therapeutic potential of exosomes, researchers employ various methods to load additional therapeutic agents into them. Drug loading involves physically or chemically incorporating small-molecule drugs or nucleic acid therapeutics into pre-isolated exosomes. Common techniques include forming transient pores in membranes via high-voltage electrical pulses, utilizing cavitation effects, repeated freeze-thaw cycles, saponin treatment, extrusion methods, and fusion with drug-loaded liposomes (Z. Chen et al., 2024b) (Haney et al., 2015). Researchers have successfully loaded Wnt signaling pathway agonists, bisphosphonate drugs, or the anti-resorptive miRNA antagonist antagomir-188 into exosomes, establishing multifunctional therapeutic systems (Y. Hu et al., 2021; Y. Wang et al., 2020). Genetic modification of source cells involves using genetic engineering techniques to overexpress specific therapeutic genes or RNA molecules in exosome-producing cells. After expression within the cells, these molecules are actively sorted and enriched within secreted exosomes. For example, transfecting plasmids overexpressing osteogenic inducers NELL-1 or BMP2 yields exosomes enriched with these pro-osteogenic proteins (F. Li et al., 2022). Similarly, stable expression of siShn3 targeting the osteogenic inhibitor Shn3 or the pro-osteogenic miR-142-5p mimetic in MSCs via lentiviral infection yields engineered exosomes with enhanced osteogenic-promoting activity (Cui et al., 2022; Li et al., 2025). Source cell pretreatment involves altering the culture environment of source cells to induce secretion of exosomes with enhanced functional properties. As previously described, hypoxia pretreatment significantly increases the levels of the proangiogenic factor VEGF and miR-126 in MSC-EVs (Han et al., 2019; W. Liu et al., 2020). Stimulating source cells with cytokines or mechanical forces can also enhance the enrichment of pro-osteogenic miRNAs or proteins in exosomes, thereby naturally boosting their therapeutic efficacy (Xiang et al., 2025; Xiao et al., 2021; Z. Zhou et al., 2023).

### 4.3 Biomaterial-based exosomes for osteoporotic fractures

For osteoporotic fractures, local application of exosomes represents a more direct and effective strategy. However, directly injected exosomes exhibit short retention times at fracture sites and are easily washed away by bodily fluids (Deng et al., 2023; X. Li et al., 2024a). Combining exosomes with biomaterials to construct controlled-release drug delivery systems can significantly enhance their local therapeutic efficacy.

Injectable hydrogels including GelMA, HAMA, and alginate hydrogel are ideal exosome carriers (S.Li et al., 2025; Yang et al., 2024a). GelMA is a photo-crosslinkable hydrogel prepared by modifying gelatin with methacrylic anhydride (MA), and its degradation rate is mainly regulated by the degree of substitution (DS) of methacrylic anhydride and crosslinking density, typically ranging from 4 to 8 weeks, which is highly consistent with the 4 to 12 week bone remodeling cycle in patients with osteoporosis (Dong et al., 2019). Gelatin exhibits excellent biocompatibility, and its degradation products can be absorbed and utilized by the body without obvious immune reactions. Moreover, its degradation process is synergistic with the sustained release behavior of exosomes (Lukin et al., 2022, Yee et al.). In the early stage (1 to 2 weeks), high-crosslinking-density GelMA hydrogel maintains structural stability and slowly releases the loaded exosomes, initiating bone repair by regulating osteoblast proliferation and osteoclast activity. In the middle stage (3 to 6 weeks), with the gradual degradation of the hydrogel, its porous structure is further opened to facilitate vascular ingrowth and cell infiltration, and the continuous release of exosomes enhances osteogenic differentiation and mineralization. In the late stage (7 to 8 weeks), the hydrogel is almost completely degraded, and new bone has been initially formed at this time, thus avoiding the interference of residual materials on bone remodeling (Xiaorong Li et al., 2024a; L. Liang et al., 2024).

For the repair of osteoporotic bone defects, GelMA is often combined with other materials to optimize its degradation rate. For example, the GelMA/HAMA composite hydrogel modulates the overall degradation cycle to weeks by virtue of the slow degradation characteristic of HAMA, which not only prolongs the exosome release window but also provides more durable mechanical support for the impaired bone repair process in osteoporotic patients (Velasco et al., 2025; Yang et al., 2024a). Studies have confirmed that after loading urine-derived stem cell exosomes, this composite hydrogel increased the BV/TV postoperatively in a rat model of osteoporotic cranial defects compared with the pure GelMA hydrogel group, and no obvious inflammatory response was induced by its degradation products, demonstrating that its degradation rate is highly matched with the demands of osteoporotic bone repair (W. Lu et al., 2023).

Alginate is a natural polysaccharide extracted from brown algae, which forms hydrogels via calcium ion crosslinking. Its degradation rate is significantly faster than that of GelMA, and it is mainly degraded by endogenous alginate lyase, making it suitable for the repair of osteoporotic fractures that require concentrated exosome release in the early stage (P. Wang et al., 2025; Ying Zhang et al., 2021c). Patients with osteoporosis need to rapidly initiate bone repair signals in the early stage of fracture. Alginate hydrogel can quickly release exosomes during degradation, and inhibit excessive osteoclast activation and promote osteoblast recruitment through early high concentrations of bioactive molecules such as osteogenesis-related miRNAs and growth factors, laying a foundation for bone repair (Yujie Zhang et al., 2021b). However, the excessively fast degradation rate of alginate may lead to insufficient exosome release after 3 weeks, and its low mechanical strength makes it difficult to meet the supportive requirements of osteoporotic fracture sites (Chen et al., 2024a). To address this, researchers have adopted two main strategies to optimize its performance: first, calcium ion gradient crosslinking or composite with HA particles to regulate the degradation rate to 3 to 6 weeks and extend the exosome release cycle (Almeida et al., 2011; Chahsetareh et al., 2024); second, composite with GelMA to form a double-network hydrogel, utilizing the moderate degradation rate of GelMA to remedy the defect of rapid degradation of alginate (Tang et al., 2022). After loading MSC exosomes, the alginate/GelMA composite hydrogel achieved an exosome release rate of 78% at 4 weeks postoperatively and a residual hydrogel content of less than 20% at 6 weeks postoperatively in a mouse model of osteoporotic femoral defects, while increasing the TMD by 27% compared with the pure alginate group, thus achieving the synergy of early signal initiation and sustained middle-stage repair (Villani et al., 2024). GelMA’s degradation rate matches bone remodeling, but its mechanical strength is insufficient for weight-bearing fracture sites. Composite with calcium phosphate ceramics can enhance mechanical support, yet this may delay hydrogel degradation and affect exosome release kinetics. Additionally, the photo-crosslinking process of GelMA may reduce exosome bioactivity. Studies have shown that UV irradiation at 365 nm for 5 min decreases the activity of osteogenic proteins such as ALP in exosomes by approximately 20% (Dong et al., 2019), which requires optimization of crosslinking parameters.

HAMA is a hydrogel prepared by the methacrylation modification of hyaluronic acid, with the slowest degradation rate (8 to 12 weeks) among the three hydrogels (Born et al., 2022). It is mainly degraded stepwise by hyaluronidase and is suitable for osteoporotic fracture scenarios requiring long-term mechanical support and continuous exosome release (Su et al., 2024). The bone repair capacity of osteoporotic patients is significantly weaker than that of healthy individuals, with their new bone formation cycle extending to 12 to 16 weeks (Singhal et al., 2018). The slow degradation characteristic of HAMA hydrogel can provide structural support throughout the repair process to avoid re-displacement of fracture sites, while continuously releasing exosomes to improve the local bone metabolic microenvironment (J. Lu et al., 2025).

To further optimize its compatibility with bone remodeling, the degradation rate of HAMA can be regulated by adjusting the degree of methacrylation (DS = 0.2**-**0.6). Low-substitution HAMA (DS = 0.2) degrades faster (8 to 10 weeks) and is suitable for patients with moderate osteoporosis; high-substitution HAMA (DS = 0.5**-**0.6) degrades more slowly (10–12 weeks), applicable for patients with severe osteoporosis or complex fracture sites (J. Lu et al., 2025). In addition, the composite of HAMA and GelMA can form a slow-medium dual-degradation-rate system (X. P. Li et al., 2021). In a model of osteoporotic vertebral compression fracture, this composite hydrogel loaded with hUCMSC exosomes achieved a vertebral height recovery rate of 83% at 12 weeks postoperatively and reduced the cortical bone porosity by 21% compared with the pure HAMA group, demonstrating that it effectively promotes bone structural regeneration in osteoporotic sites through the precise regulation of degradation rate (J. Lu et al., 2025).

3D-printed scaffolds can be manufactured using 3D printing technology to create calcium phosphate bioceramic scaffolds with biomimetic trabecular structures and controllable porosity. These scaffolds not only provide excellent mechanical support, but their porous structure also mimics the extracellular matrix of bone, creating a favorable microenvironment for bone cell adhesion, proliferation, and ingrowth. Loading exosomes onto the scaffold surface or within its interior enables the gradual release of exosomes during scaffold degradation, synergistically enhancing osteogenic differentiation and new bone formation (J. Zhang et al., 2016). Biodegradable films/coatings are critical for the rapid and stable osseointegration of orthopedic implants with bone tissue, ensuring surgical success. In patients with osteoporosis, osseointegration capacity is often compromised, leading to implant loosening and increased revision rates. Human adipose-derived stem cell (hASC)- derived exosomes were immobilized on a poly (lactic-co-glycolic acid) (PLGA) scaffold coated with polydopamine (PLGA/pDA). This system was demonstrated to sustain slow-release of exosomes, significantly enhancing bone regeneration. This effect was achieved at least partially through bone-inducing effects and by promoting mesenchymal stem cell migration and homing (W. Li et al., 2018). Exosome-functionalized PLGA/Mg-GA MOF scaffolds. This composite scaffold enables sustained release of exosomes, magnesium ions, and gallic acid, demonstrating excellent biocompatibility, osteogenic, pro-angiogenic, and anti-inflammatory activities in both *in vitro* and *in vivo* rat cranial defect models (Kang et al., 2022). PLGA and PEG triblock copolymer microspheres were employed to enhance exosome encapsulation and controlled release. Integrated into a three-dimensional tissue engineering scaffold, the controlled release of osteogenic exosomes recruited endogenous cells and promoted their differentiation, thereby accelerating bone healing in mouse cranial defects without exogenous cell transplantation (Swanson et al., 2020). PLGA nanoparticles encapsulating human urine-derived stem cell-derived extracellular vesicles prevent and treat periprosthetic bone resorption through miR-21-5p-mediated anti-inflammatory mechanisms. This offers a novel potential therapeutic strategy for preventing implant loosening, a late-stage complication (Yinan Wang et al., 2024).

## 5 Conclusion

Exosomes as key carriers of intercellular signaling, play a crucial role in maintaining bone homeostasis through the diverse bioactive molecules they transport, including proteins, miRNAs, and circRNAs. They profoundly influence the dynamic equilibrium between bone formation and resorption, thereby participating in the onset and progression of osteoporosis. Exosomes derived from mesenchymal stem cells, young individuals’ plasma, specific plants, or probiotics typically exert protective effects by promoting bone formation and inhibiting bone resorption, offering valuable natural therapeutic resources for osteoporosis. Conversely, exosomes originating from osteoclasts, M1 macrophages, and tumor cells often carry signals that promote bone resorption or suppress bone formation, accelerating bone mass loss. Based on these mechanisms, exosomes hold broad application prospects in the field of osteoporosis. In diagnostics, their use as biomarkers in liquid biopsies enables early, non-invasive, and precise disease detection. Therapeutically, exosomes demonstrate unparalleled advantages over traditional drugs, whether as natural therapeutic agents or engineered smart drug delivery systems. Particularly when combined with advanced biomaterials technology, they offer powerful innovative strategies for overcoming challenging osteoporotic fractures.

Despite explosive growth in exosome research over the past decade, the path from laboratory to clinical application remains fraught with challenges. Currently, there is no universally accepted, unified gold standard for exosome isolation and purification. Exosomes obtained through different methods exhibit significant variations in purity, yield, and subpopulation distribution. This severely impacts the reproducibility of research findings and the ability to compare results across studies, presenting a primary technical hurdle that must be resolved before clinical translation. The contents of exosomes are exceptionally complex, and their regulation of bone metabolism typically results from the synergistic actions of multiple molecules. Current research predominantly focuses on single or a few key molecules, leaving our understanding of how multiple molecules within exosomes form intricate regulatory networks and their overall effects largely incomplete.

Although natural exosomes exhibit low immunogenicity, the long-term safety, immunogenicity risks, and metabolic pathways of engineered exosomes require systematic and rigorous evaluation in large animal models that more closely mimic human physiology. While numerous exosomal molecules demonstrate potential as diagnostic biomarkers, most studies remain confined to small-sample, retrospective case-control investigations. The clinical translation of exosomes in bone diseases such as osteoporosis also faces prominent regulatory hurdles and deficiencies in Good Manufacturing Practice (GMP) standardization, which are two critical gaps not fully addressed in current research (C. Y. Ma et al., 2024; Tajafrooz et al., 2025). Despite exosomes unique advantages in bone disease treatment, including targeted delivery, e.g., CXCR4-modified exosomes homing to bone marrow, CAP-Nrf2-Exos targeting chondrocytes, and regulation of osteogenic differentiation via pathways such as miR-29a-3p/NFIA/Wnt and miR-378a-3p-inhibited NF-κB-MAPK, the core bottlenecks lie in ambiguous regulatory classification and inadequate GMP process controllability (Q. Li et al., 2025; Verma and Arora, 2025). From a regulatory perspective, due to differences in exosome sources e.g., MSCs, macrophages, body fluids after traditional Chinese medicine intervention and modification degrees, there is a lack of unified classification standards (Cheng and Kalluri, 2023; Tandon and Srivastava, 2025). Key Quality Attributes (CQAs) specific to bone diseases e.g., bone-targeting efficiency, osteogenic cargo stability remain undefined, and there are significant gaps in safety monitoring data, including immunogenicity, off-target accumulation, and long-term impacts on bone metabolism (W. Hu et al., 2025; Lee et al., 2026). At the GMP level, raw material quality control needs to be strengthened with rigorous donor screening and genetic stability verification, e.g., STR typing of MSCs, quantitative detection of active components in traditional Chinese medicine extracts (Weon et al., 2014); The production process lacks automated separation technologies and standardized procedures, leading to poor batch consistency; quality control (QC) requires a combination of multiple techniques to quantify intact exosome yield, bone-specific cargo activity, and osteogenic differentiation-inducing bioactivity (Murgia et al., 2016; Novoa et al., 2024).

To resolve the lack of unified standards for exosome isolation, international academic institutions and regulatory bodies should collaborate to establish bone disease-specific technical guidelines. For basic research and clinical trials, a combination of SEC and immunoaffinity capture is recommended as a preferred isolation strategy to balance purity and yield. For QC, CQAs should include: physical properties (particle size distribution: 40–150 nm, zeta potential: -20 to -10 mV); biological activity (osteogenic differentiation-inducing capacity of MSCs significantly higher than control group, detected via ALP staining or alizarin red staining); safety indicators (endotoxin content < 0.5 EU/mL, protein contamination < 10% of total exosome cargo). Additionally, automated isolation technologies such as tangential flow filtration should be promoted to improve batch consistency, with the goal of reducing inter-batch variation in osteogenic cargo content to less than 20%.

To overcome the fragmentation of mechanistic research, multi-omics integration strategies including single-cell RNA sequencing and proteomics should be adopted to construct “exosome cargo-bone cell-bone microenvironment” regulatory networks. For example, combining CRISPR-Cas9 screening with exosome cargo profiling to identify key synergistic molecule pairs including miR-26a-5p and CTHRC1 in urine-derived stem cell exosomes.

To enhance the clinical applicability of engineered exosomes: for targeting modification, natural bone-targeting peptides such as SDSSD should be prioritized over chemical conjugation such as bisphosphonate to reduce potential cytotoxicity, with modification density optimized based on preclinical data avoiding over-modification that impairs exosome bioactivity; for genetic engineering, inducible promoter systems such as Tet-On/Tet-Off are recommended to achieve transient and controllable expression of targeting proteins such as CXCR4, avoiding long-term interference with the bone marrow microenvironment; long-term safety evaluation in large animal models such as cynomolgus monkeys should focus on immunogenicity and off-target accumulation.

Regulatory authorities should clarify the classification of exosome-based products: naturally derived exosomes such as MSC-EVs can be categorized as biological products, while engineered exosomes such as bone-targeted peptide-modified exosomes may be classified as combination products of drugs and medical devices. For GMP production: raw material control should include strict donor screening such as STR typing of MSCs, detection of common pathogenic microorganisms and quantitative analysis of key active components such as osteogenic miRNA content; production processes should adopt closed automated systems to reduce human contamination, with key steps such as exosome loading of therapeutic agents validated for reproducibility; QC should integrate multiple detection techniques nanoparticle tracking analysis, Western blot for CD9/CD63/CD81, and functional assays for osteogenic activity to ensure product quality consistency.

Future clinical trials should adopt a stratified design based on osteoporosis subtypes postmenopausal, diabetic, glucocorticoid-induced and patient characteristics age, gender, bone mineral density T-score. For phase I trials, dose escalation should be guided by bioactivity rather than concentration, with the starting dose determined by preclinical data. Phase II/III trials should focus on key endpoints including fracture risk reduction, bone mineral density improvement assessed via DXA, and long-term safety. Additionally, combination therapies such as exosome-functionalized hydrogels + low-dose bisphosphonates can be explored to achieve synergistic effects and reduce the dosage of exosomes.

Only through the synergistic strategy of clear regulatory classification, strict GMP process control, and clinical focus on long-term safety can the core challenges of controllability, consistency, and safety be resolved, unlocking exosomes full application potential in bone disease treatment.
